# Overexpression of proinflammatory cytokines in dental pulp tissue and distinct bacterial microbiota in carious teeth of Mexican Individuals

**DOI:** 10.3389/fcimb.2022.958722

**Published:** 2022-12-08

**Authors:** Ana Pamela Gómez-García, Yolanda López-Vidal, Sandra Pinto-Cardoso, María Magdalena Aguirre-García

**Affiliations:** ^1^ Unidad de Investigación UNAM-INC, División de Investigación, Facultad de Medicina, UNAM, Instituto Nacional de Cardiología Ignacio Chávez, Ciudad de México, México; ^2^ Programa de Inmunología Molecular Microbiana, Departamento de Microbiología y Parasitología, Facultad de Medicina, Universidad Nacional Autónoma de Mexico, Ciudad de México, México; ^3^ Centro de Investigación en Enfermedades Infecciosas, Instituto Nacional de Enfermedades Respiratorias Ismael Cosío Villegas, Ciudad de México, México

**Keywords:** dental pulp tissue, Toll-like receptors, proinflammatory cytokines, human oral microbiome, caries, oral microbiota, dental plaque

## Abstract

The prevalence of dental caries in the Mexican adult population aged 20 to 85 years is around 93.3%, and 50% in Mexican children and adolescents. Worldwide, it is the most common non-communicable disease. One of the main etiological factors for dental caries is the oral microbiome and changes in its structure and function, with an expansion of pathogenic bacteria like *Streptococcus mutans*. The exposed dental pulp tissue triggers an innate immune response to counteract this bacterial invasion. The relation between oral dysbiosis and innate immune responses remains unclear. We aimed to understand the relationship between innate immune response and the oral microbiota by quantifying the expression of Toll-like receptors (TLRs) and proinflammatory markers (cytokines and a chemokine) in dental pulp tissue, either exposed or not to carious dentin, and to correlate this information with the oral microbiome found in healthy teeth and those with moderate caries. RNA was purified from pulp tissue, subjected to RT-qPCR and analysed with the ^ΔΔCt^ method. Supragingival dental plaque of non-carious teeth and dentin of carious teeth were subjected to 16S targeted sequencing. Principal coordinate analysis, permutational multivariate ANOVA, and linear discriminant analysis were used to assess differences between non-carious and carious teeth. Correlations were assessed with Spearman´s test and corrected for multiple comparisons using the FDR method. The relative abundance (RA) of *Lactobacillus, Actinomyces, Prevotella*, and *Mitsuokella* was increased in carious teeth; while the RA of *Haemophilus* and *Porphyromonas* decreased. *Olsenella* and *Parascardovia* were only detected in carious teeth. Significant overexpression of interleukin 1 beta (IL1 β), IL6, and CXCL8 was detected in pulp tissue exposed to carious dentin. IL1β correlated positively with TLR2 and *Actinomyces*; yet negatively with *Porphyromonas.* These findings suggest that immune response of pulp tissue chronically exposed to cariogenic microbiome is triggered by proinflammatory cytokines IL1β and IL6 and the chemokine CXCL8.

## 1 Introduction

According to the World Health Organization (WHO), approximately 3.58 billion people worldwide are affected by oral diseases; and the most prevalent non-communicable disease (NCD) worldwide is dental caries, otherwise known as tooth decay, followed by periodontal disease (James et al., 2018). In México, the National System for Epidemiological Surveillance of Buccal Pathologies (SIVEPAB, 2019) reported that caries affects 93.3% of the Mexican population aged between 20 and 85 years and around 50% of children and adolescents (, 2019). When left untreated, these two diseases (caries and periodontal disease) can lead to dental pulp tissue damage, infection in the dental pulp tissue, tooth abscess, and eventually tooth loss. Therefore, these two diseases are major public health problems (Liu et al., 2020). Furthermore, oral health has been at the forefront of research and intervention therapeutics as it is linked to systematic diseases (Peng et al., 2022). Considering that the etiology of dental caries is multifactorial, recent data have implicated the oral microbiome as one of the main etiological factors in the development of oral diseases. The human oral microbiome consists of a well-organized collective of microorganisms, the vast majority of which are commensals. The extended human oral microbiome database (eHOMD, April 2022) lists 774 bacterial species in the oral cavity, divided into different phyla such as *Firmicutes*, *Actinobacteria*, *Proteobacteria*, *Bacteroidetes*, *Synergistetes*, and *Proteobacteria* (Escapa et al., 2018). *Streptococcus viridians* (formerly known as S. *mitis*) is the predominant bacterial species in the human oral cavity, found in saliva and dental plaque (among other niches). *S. oralis* is the initial colonizing species of the tooth surface and may regulate the first events in the formation of dental biofilm (Zbinden et al., 2015). The pathogenesis of caries is associated with aberrant changes in the oral microbiome, often termed dysbiosis, with the prevalence of bacteria, collectively known as cariogenic microbiota such as *S. mutans* (Takahashi and Nyvad, 2011; Belstrøm et al., 2014; Bezerra et al., 2016; Fakhruddin et al., 2019).

In recent years, molecular studies have unraveled how genera like *Lactobacilli, Actinomyces, Bifidobacterium, Veillonella, Cutibacterium* (formerly *Propionibacterium*) and *Atopobium* might play significant roles in the pathology of dental caries, acting synergistically with or antagonistically against *Streptococcus mutans* (Aas et al., 2008; Wolff et al., 2013; Wolff et al., 2019). Several authors have reported bacteria associated with caries in adults from different ethnic groups: Munson et al. (2004) reported bacterial species associated with middle and advancing front of dental caries showing a diverse bacterial community, including *S. mutans, Lactobacillus, Rothia dentocariosa*, and *Propionibacterium.* Chhour et al. (2005) described the microbial diversity in advanced caries, and reported an increased abundance of *Lactobacillus, Prevotella, Selenomonas, Dialister, Fusobacterium, Eubacterium, Olsenella, Bifidobacterium, Propionibacterium*, and *Pseudoramibacter*. *S. mutans* was not detected in this study. Other research groups detected an increase in *Propionibacterium acidifaciens*, this latter being approximately 40-fold more abundant than *S. mutans* in deep caries samples (Wolff et al., 2013). Today, researchers widely agree that *S. mutans* serve as a good marker for dental caries, but not necessarily as the only and exclusive etiologic agent (Beighton, 2005). These findings concur with earlier studies, demonstrating that there is a distinctive microbiome of the healthy oral cavity that is different from that associated with caries (Aas et al., 2008) yet, the composition of the oral microbiome in moderate caries, a stage characterized by enamel breakdown, without visual signs of dentin exposure, has been scarcely reported.

Over time, dental caries lead to an irreversible disease of the calcified tissue of the tooth, by demineralization and subsequent destruction of the organic substance of the tooth, resulting in progressive tooth decay leading to cavitation (Mazzoni et al., 2015). As enamel demineralization continues, dentin is exposed to bacterial invasion, resulting in further demineralization and cavitation (Takahashi and Nyvad, 2016). If caries remains untreated, they turn to deep caries that penetrate the entire thickness of the dentin with specific pulp exposure. When dental pulp cells are exposed to dental caries, they respond directly by expressing various chemokines and cytokines to promote cellular defence processes and attempt to repair (Farges and Lyon, 2015). There are no symptoms or visual signs in the initial stages of caries. Symptoms begin when the carious lesions grow and progress to the dentin (Selwitz, 2007). At this stage, an inflammatory response occurs in the dental pulp, along with pulp tissue ischemia with severe pain (Kwack and Lee, 2022).

Bacterial colonization of the carious dentin can be impeded by the innate immune response of exposed dental pulp tissue. The initial innate immune response to cariogenic bacteria and their components is triggered by the overexpression of cell receptors of the pulp-dentin complex, leading to the expression of regulatory and proinflammatory cytokines (Hahn and Liewehr, 2007a). The initial steps are difficult to define because dental decay is usually characterized by a slow progression, which takes place in a vertical direction from the tooth surface to the dental pulp tissue. Since dental pulp tissue is exposed to an abundance of antigenic components when a carious lesion reaches 2 mm (Hahn and Liewehr, 2007a), it can become irreversibly inflamed; at this point, an adaptive immune response is triggered (Hahn and Liewehr, 2007b). The abundance of antigenic components typically causes pulp infections that can induce pulp necrosis and apical periodontitis in severe cases characterized by the destruction of periodontal bone (Sci-Hub; Liu et al., 2020; Pourhajibagher et al., 2022). Consequently, oral microorganisms can translocate to other organs (Zarco et al., 2012; Camelo-Castillo et al., 2015; Yamashita and Takeshita, 2017; Gao et al., 2018; Nørskov-Lauritsen et al., 2019).

To have a better understanding on how exposed human dental pulp tissue responds to cariogenic microbiota in a moderate degree of dentin caries, it would be best to evaluate changes in microbial diversity and community structure that accompany tooth decay as well as *in situ* immune responses of the pulp tissue. One avenue to investigate both microbial communities and immune response under natural conditions is to examine teeth extracted from individuals with caries. Numerous studies have reported microbial community changes and immune responses in caries (Mutoh et al., 2007; Farges et al., 2011; Simón-Soro et al., 2013; Belstrøm et al., 2014). The aims of this study were to characterize the oral microbiome associated with caries of permanent teeth in adults, to describe bacterial profiles associated with the caries-free and active-caries teeth, to quantify the expression of TLRs and inflammatory markers in the dental pulp tissue, and to explore relationships between the oral microbiota and markers associated with innate immune responses.

## 2 Materials and methods

### 2.1 Study design

This study was conducted according to the World Medical Association (WMA) Declaration of Helsinki (Simón-Soro et al., 2013) on experimentation involving human subjects and was approved by the Ethics in Research Committee of the Faculty of Medicine of the UNAM, under project approval registry FM/DI/030/SR/2019.

### 2.2 Study subjects

Adult subjects were recruited consecutively from the maxillofacial surgery clinics of the Faculty of Dentistry of the Universidad Nacional Autónoma de México (UNAM). Subjects were invited to participate. If they accepted, teeth were extracted due to clinical indications other than orthodontic reasons or dental prosthetic rehabilitation. All participants were informed about the study´s objectives, and signed an “Informed and Free Consent Form”, as required by the Ethics in Research Committee.

### 2.3 Eligibility criteria

#### 2.3.1 Subjects

Subjects who met the following criteria were enrolled in the study: subjects ≥18 years of age, both sexes, referred to the maxillofacial surgery clinics and who voluntarily accepted to donate extracted teeth. Exclusion criteria included pregnancy, breastfeeding, smoking, periodontal disease, any systemic disease preventing enrollment, use of systemic antibiotics in the previous three months, current or previous periodontal treatment, and current orthodontic therapy.

#### 2.3.2 Teeth

Teeth (also known as dental organs) who met the following criteria were used: freshly (up to 24 hours) extracted, vital, permanent, mature, anterior and/or posterior [bicuspid (also known as premolars) and molars] teeth, exhibiting either caries-free, inactive or primary active moderated caries involving the dentin tissue. Exclusion criteria: any definitive or temporary dental fillings, restorative treatments, dental pulp tissue exposition, necrotic pulp tissue, chronic apical periodontitis, degrees of caries except moderate caries extending to the dentin tissue.

### 2.4 Samples

#### 2.4.1 Teeth

According to the eligibility criteria, thirty-one teeth, bicuspid and molars, were collected and stored in a new sterile recipient containing a sterile physiological solution. Teeth were classified into two groups, non-carious (n = 14) or carious (n = 17) teeth. Extracted teeth were transported on ice to the Unidad de Investigación UNAM-INC. Samples were processed immediately or stored at - 20°C. Collection, classification and processing of all teeth were carried out by a dentist clinician (AG-G) trained in identifying visible caries lesions.

#### 2.4.2 Supragingival dental plaque

All samples (SDP and carious dentin tissue) were processed by AG-G trained. SDP samples were collected before dental extraction. Subjects were asked to abstain from oral hygiene: tooth brushing, flossing, mouth rinsing and chewing gum consumption for at least 24 hours before SDP sampling. Before SDP sampling, cotton rolls were placed in the oral cavity to avoid saliva contamination. Using an autoclaved sterile Hu-Friedy dentin excavator (Hu-Friedy^©^ Mfg. Co., LLC, Chicago; IL, Unites States), SDP samples were scraped off at the vestibular side of teeth within the same quadrant (left or right) of the upper/lower maxilla (Aas et al., 2008; Simón-Soro et al., 2013). Samples were stored in new sterile Eppendorf tubes free from RNases/DNases containing 500μl of 70% ethanol (Sigma-Aldrich^®^ Co. LLC, Saint Louis, MO, US) and shipped to the Unidad de Investigación UNAM-INC for further assays. Samples were processed immediately, or stored at - 20°C.

#### 2.4.3 Dental pulp tissue

All samples were processed by AG-G at Unidad de Investigación UNAM-INC and extracted under sterile conditions. To collect DPT from carious and non-carious teeth, a longitudinal furrow was made along the surface of each tooth by using a diamond disc revolving at a low velocity and irrigating with a sterile physiological solution. Subsequently, teeth were sectioned with an autoclaved sterile hammer and chiseled to expose the DPT. Then, the DPT was gently removed with a sterile and very sharp Hu-Friedy dentin spoon excavator and sterile Hu-Friedy dental tweezers. The tissue was macerated with a sterile pestle and embedded in TRIzol^®^ (Life Technologies, San Diego, CA, US).

#### 2.4.4 Carious dentin tissue

After dental extractions, teeth were shipped to the Unidad de Investigación UNAM-INC and proceeded by AG-G to collect DT from individual carious lesions. Caries-free stage, or degrees of inactive caries and moderate caries were confirmed by visual examination. Moderate caries was defined as carious lesions extending to the outer or middle third of dentin tissue (Couve et al., 2014). DT was extracted by scraping the tissue with an autoclaved sterile dentin excavator (Hu-Friedy^©^ Mfg. Co., LLC, Chicago; IL, Unites States) or pulverizing the decaying surface with an autoclaved sterile endodontic file and ultrasound under sterile conditions (Simón-Soro et al., 2013; Wolff et al., 2019). Samples were placed separately in new sterile Eppendorf tubes free of RNases/DNases containing 500μl of 70% ethanol. Samples were processed immediately or stored at - 20°C.

### 2.5 RNA extraction from dental pulp tissue

Total RNA was isolated with TRIzol^®^ reactive (Life Technologies, San Diego, CA, US), according to the manufacturer’s instructions and as described by *Chomczynski* (1993) (Mazzoni et al., 2015). The pellet was suspended in molecular biology grade sterile water, and the concentration and purity of the RNA were determined using the NanoDrop^™^ 2000c spectrophotometer (ThermoFisher Scientific Waltham, MA, US), considering absorbance at A260/280 ≥1.8. Total RNA integrity was verified by electrophoresis in 1% agarose gel stained with ethidium bromide and visualized in a UV ChemiDoc MP™ transilluminator (Bio-Rad Hercules, CA, US). Gel images were taken with a digital camera. RNA integrity was confirmed by capillary electrophoresis on an Agilent 2100 bioanalyzer (Santa Clara, CA, US), considering an integrity value of RNA (RIN≥6.0).

#### 2.5.1 Reverse transcription-qPCR

Five hundred ng of RNA was treated with DNase (2 U/μl) TURBO DNA-free™ kit (Invitrogen Corp., Carlsbad, CA, US). Next, reverse transcription of 100ng of RNA was carried out with the Superscript II™ Reverse Transcriptase kit (Invitrogen Corp., Carlsbad, CA, US), using the Applied Biosystems 2720 thermal cycler (Applied Biosystems LLC, Bedford, MA, US) with an initial step at 65°C for 5 min, followed by a reverse transcription at 42°C for 52 min, and a final inactivation at 70°C for 15 min. Complementary DNA (cDNA) was stored at -20°C until further use.

#### 2.5.2 Real-time polymerase chain reaction

Messenger RNA (mRNA) was amplified with specific primers for each marker (**Table S1**) using the NZY qPCR Green Master mix 2X kit (NZYtech Lisbon, Portugal). Markers were Toll-Like Receptor 2 (TLR2), and TLR4, tumor necrosis factor-alpha (TNFα), interleukin 1 beta (IL1β), IL6, chemokine C-X-C motif chemokine ligand 8 (CXCL8, also known as IL8), IL10, and transforming growth factor beta (TGFβ). Amplification was performed with the following conditions: denaturalization at 94°C for 15 sec, alignment at 57°C for 30 sec, and an extension at 72°C for 45 sec, for a total of 34 cycles. After amplification, the specificity of each PCR amplicon was determined by analyzing the melting curve and visualizing all amplicons in 2% agarose gel. Each sample was read in duplicate. Three internal controls were used: human macrophages stimulated with lipopolysaccharide (Mφ/LPS), without the cDNA mold (NTC), and without the transcriptase enzyme (NRT). Data was acquired and examined on a CFX96™ Real-Time thermal cycler Bio-Rad device (Bio-Rad, Hercules, CA, US). Results were reported with the 2^-ΔΔCt^ method and normalized with the endogenous beta-actin genes and glyceraldehyde-3-phosphate dehydrogenase (GAPDH).

### 2.6 DNA extraction from supragingival dental plaque and carious dentin tissue

DNA was extracted from the supragingival dental plaque and carious dentin tissue with the QIamp^®^ DNA mini kit (Qiagen Inc., Valencia, CA, USA). Additionally, RNA was removed by Rnase (10mg/ml) digestion. DNA concentration and purity were measured in triplicate using the NanoDrop 2000c apparatus (ThermoFisher Scientific, Carlsbad, CA, USA), and samples with an A260/280 ≥1.8 were used. The mean value was calculated for each sample. Subsequently, the integrity of the DNA was examined by electrophoresis in 1% agarose gel stained with ethidium bromide and visualized in a UV ChemiDoc MP™ Bio-Rad transilluminator.

#### 2.6.1 16S rRNA gene amplicon sequencing

DNA samples meeting quality control requirements were sent to Macrogen Co., LTD (Gasan-dong, Seoul, South Korea) for high-throughput amplicon sequencing of the V3 – V4 regions of the 16S rRNA gene on the Illumina MiSeq, Paired-end 2 × 300 bp, system (Illumina, San Diego, CA, USA). The primers used were: 341F (CTT ACG GGN GGC WGC AG) and 805R (GAC TAC HVG GGT ATC TAA TCC) (Herlemann et al., 2011). PCR amplification, libraries and sequencing were performed according to Macrogen´s Protocols. All samples were sequenced in the same sequencing run.

#### 2.6.2 Bioinformatic analysis of 16S rRNA gene

Raw demultiplexed paired sequences were imported into QIIME2-2020.8 (Quantitative Insights into Microbial Ecology 2) (Bolyen et al., 2019). First, primers were removed with q2-cutadapt (Martin), next demultiplexed sequences were filtered, denoised, merged and trimmed in the region flanked by sequencing primers, 341F and 805R (Herlemann et al., 2011; Bach et al., 2019; Kawamoto et al., 2021). Forward and reverse reads were trimmed at position 260 and 210 respectively, and merged with a minimum overlap of 12 bp with q2-dada2 (Callahan et al., 2016) to obtain amplicon sequence variants (ASVs). Taxonomy was assigned with q2-feature‐classifier (Bokulich et al., 2018) by using a trained naïve Bayes taxonomy classifier for the V3 and V4 regions of the 16S rRNA gene, the trained reference was the eHOMD 15.2 database at 99% identity (Escapa I et al., 2020). The ASV table was filtered to remove ASVs not present in at least two samples with q2-feature-table (Weiss et al., 2017). Alpha diversity metrics (observed species, Chao1, Shannon & Simpson), beta diversity metric (Bray‐Curtis dissimilarity), and principal coordinates analysis were performed using q2-diversity after rarefying all samples to 10,000 sequences per sample using q2-feature-table rarefy, see **Figure S1** (Weiss et al., 2017).

### 2.7 Statistical analysis

Data were processed with the Statistical Package for Social Sciences (SPSS, version v21), GraphPad Prism v8 (La Jolla, CA, USA) and RStudio 3.6.3. Normal distribution was assessed with the Shapiro-Wilk test. If assumption of normality was confirmed, the parametric unpaired Student’s t-test for independent samples was used, assuming equal variances. If assumption of normality was not confirmed, the nonparametric Mann-Whitney U-test for the comparison of two unpaired groups and the nonparametric Spearman’s rank correlation coefficient, correcting for multiple comparisons using the false discovery rate (FDR) method. For nominal and dichotomic data, the chi-squared test or the Fisher´s exact test was used for categorical variables as appropriate. Alpha diversity metrics, the relative abundance at phylum and genus level, and gene expression were compared between non-carious and carious using the Mann-Whitney *U* test. Also, to identify discriminant features between groups, linear Discriminant Analysis Effect Size (LEfSe) was conducted using the default parameters (Segata et al., 2011). For the beta diversity metric, the permutational multivariate analysis of variance (PERMANOVA) test was used. Statistical significance was set at a *p ≤* 0.05.

## 3 Results

### 3.1 Study subjects

Demographic characteristics of study subjects are summarized in **Table 1**. Teeth were donated by 31 participants, 20 (64.5%) women and 11 (35.5%) men. The average age of our cohort was 28.5 ± 5.6 years old. Subjects were classified into two groups, according to the presence of caries, 14 (45.2%) had no caries or inactive caries (non-carious subjects) and 17 (54.8%) had caries (carious subjects). Two (6.5%) individuals had a history of never having had caries (caries free), 12 (38.7%) had a history of caries (inactive caries), and 17 (54.8%) suffered from active caries. No significant difference was observed between these 2 groups for sex and age, however, a difference was observed in the presence of caries, as expected (*p <* 0.001).

**Table 1:** Demographic characteristics of the donors of teeth.

	Non-carious	Carious	Overall	*p*
**Sample size**	14 (45.2)	17 (54.8)	31 (100)	
**Sex** ^b^				0.258
Female	11 (35.5)	9 (29.0)	20 (64.5)	
Male	3 (9.7)	8 (25.8)	11 (35.5)	
**Age, years** ^a^				0.210
17 - 22	3 (9.7)	2 (5.9)	5 (15.5)	
23 - 27	4 (12.9)	3 (9.7)	7 (22.6)	
27 - 32	5 (16.1)	5 (16.1)	10 (32.2)	
33 - 37	2 (6.5)	6 (19.4)	8 (25.9)	
38 - 43		1 (3.2)	1 (3.2)	
	27.1 ± 4.9 27.5 [9.8]	29.7 ± 6.0 29.0 [6.5]	28.5 ± 5.6 28.0 [8.0]	
**Antecedents of caries** ^**b**^				< 0.001***
Caries-free	2 (6.5)		2 (6.5)	
Inactive caries	12 (38.7)		12 (38.7)	
Active caries		17 (54.8)	17 (54.8)	

Data are shown as the percentage of the total number of tooth donors; M ± SD, mean ± standard deviation, otherwise indicated as the median and interquartile range [IQR].

a Parametric unpaired Student’s t-test for independent samples assuming equal variances. For nominal and dichotomic variables with expected values < 5.

b Fisher exact test was applied. Statistical significance was considered at 95% of confidence (*p* ≤ 0.05). ***p < 0.001.

### 3.2 Oral bacterial microbiota associated with moderate caries

**Figure 1:**
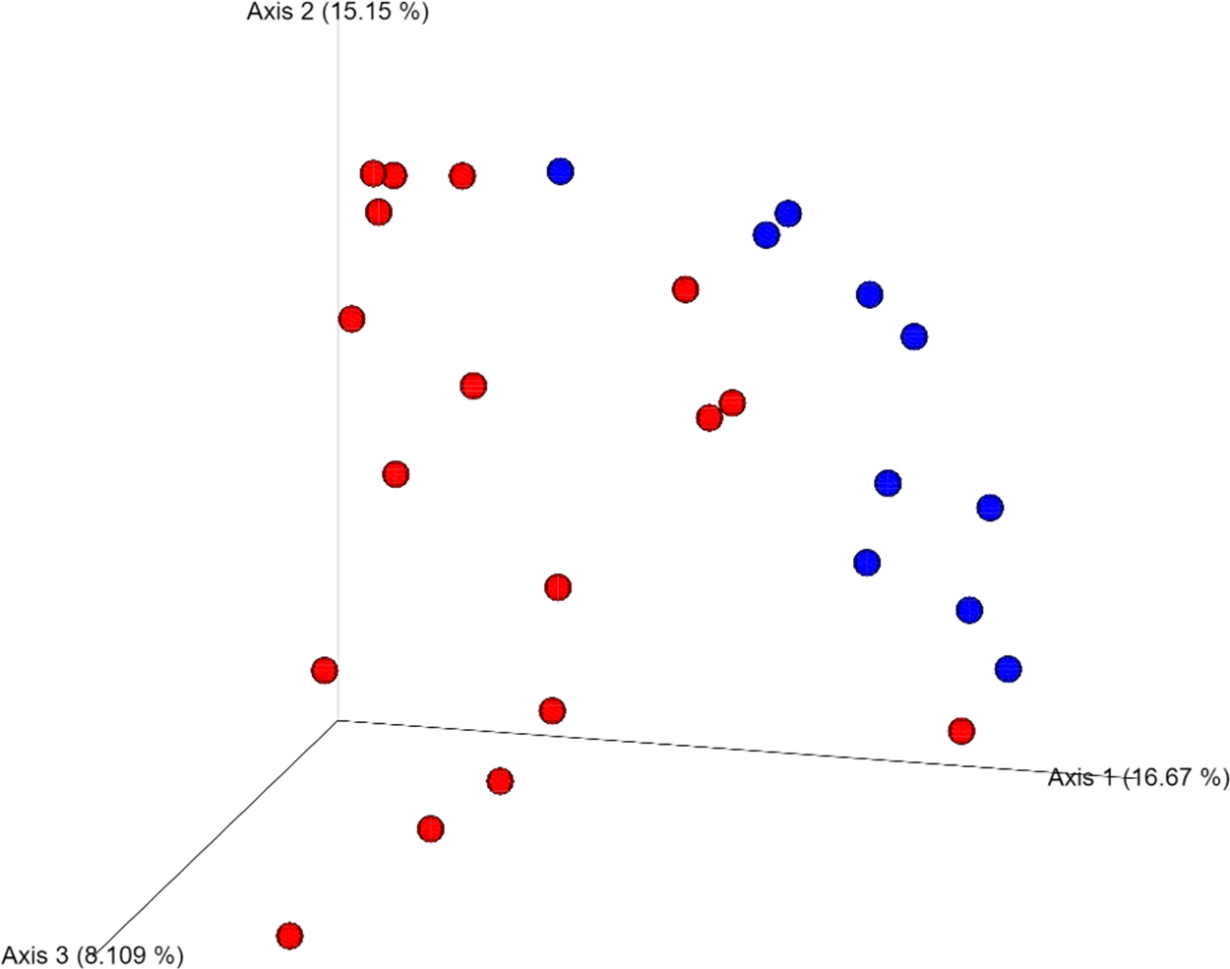
Beta diversity analysis of the two groups: non-carious and carious teeth. Bacterial communities were visualized using principal coordinate analysis (PCoA) and Bray-Curtis dissimilarity index. Permutational multivariate analysis of variance (PERMANOVA) was used to assess differences between the two groups (PERMANOVA; *p =* 0.001). Blue dots refer non-carious group and red dots refer carious group.

**Figure 2:**
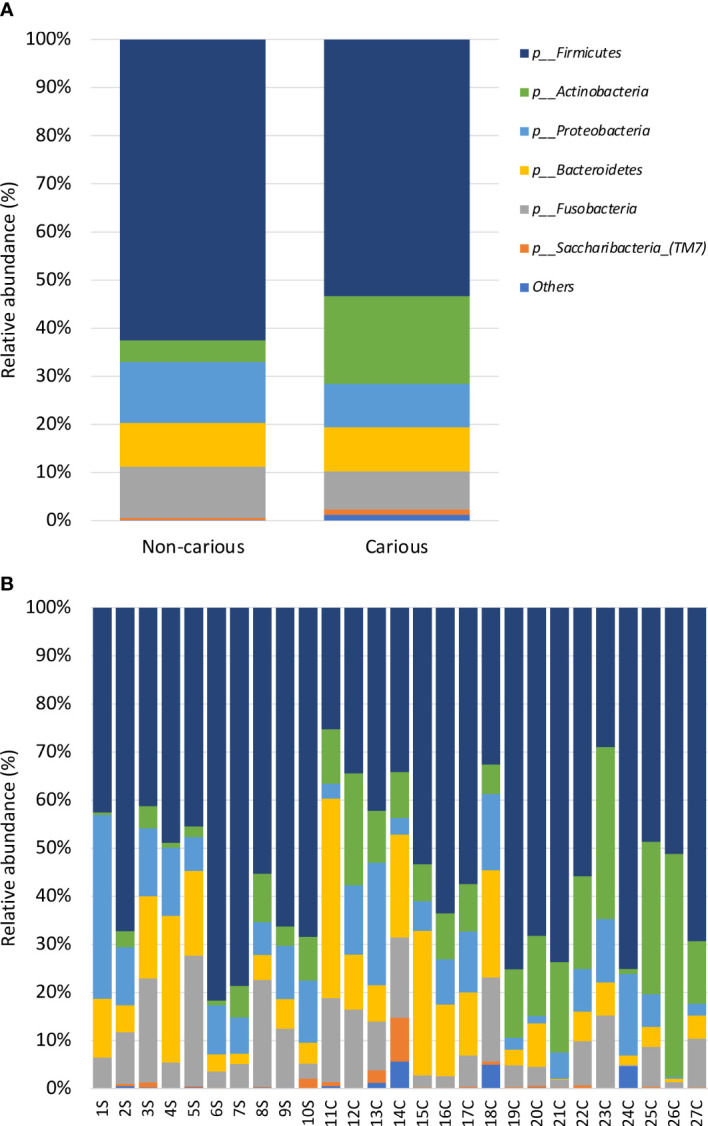
Relative abundance by phyla in the oral bacterial microbiota. Taxonomic barplots showing the top 11 phyla found in the oral microbiota **(A)** faceted by group: non-carious and carious teeth **(B)** of everyone. The letter *S* refers to samples taken from the supragingival dental plaque of non-carious teeth; the letter *C* refers to samples taken from the infected dentin tissue of moderate caries.

**Figure 3:**
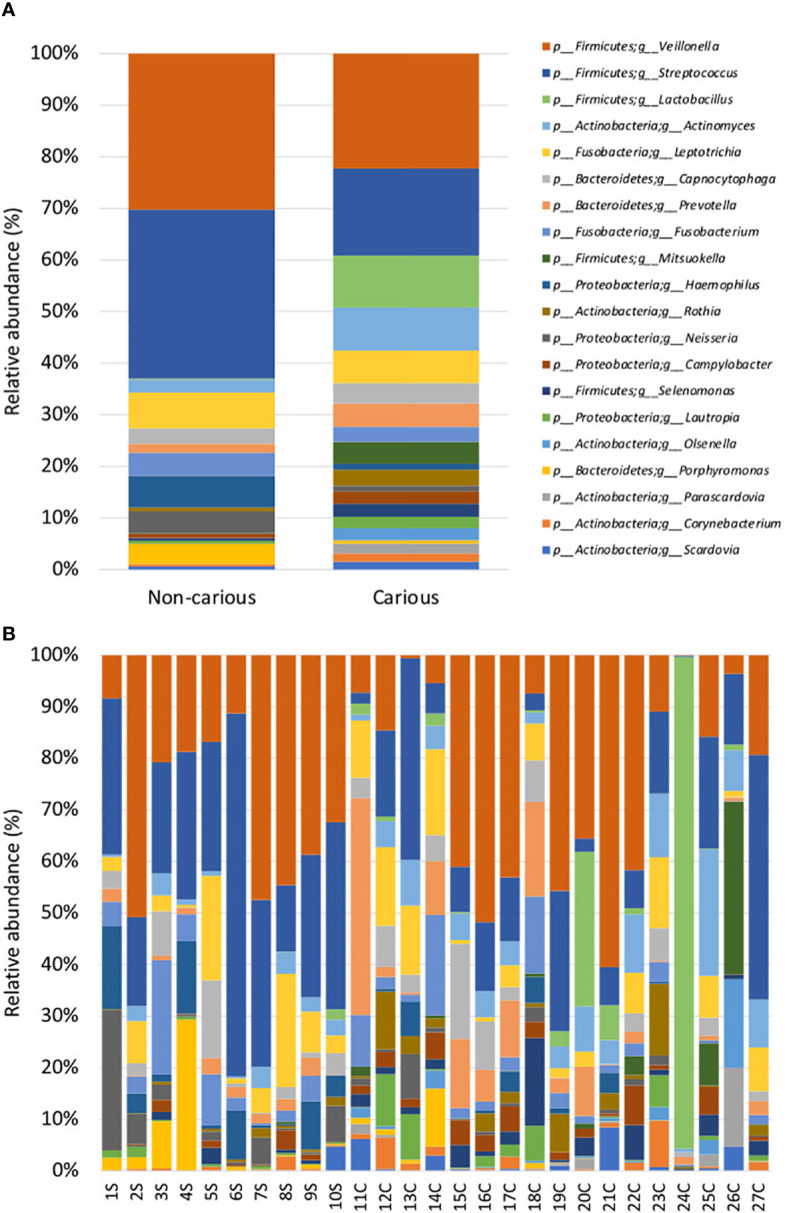
Relative abundance by genus in the oral bacterial microbiota. Taxonomic barplots showing the top 20 genera found in the oral microbiota **(A)** faceted by group: non-carious and carious teeth **(B)** of everyone. The letter *S* refers to samples taken from the supragingival dental plaque of non-carious teeth; the letter *C* refers to samples taken from the infected dentin tissue of moderate caries.

Four DNA samples from the non-carious group did not fulfil the quality control criteria established by Macrogen Co. and were not sequenced. A total of 2,515,688 raw sequences were obtained from DNA isolated from supragingival dental plaque (n = 10) and carious dentin tissue (n = 17), with a mean of 93,173.6 sequences per sample. After quality control, a total of 1,035,182 sequences remained, with a mean of 38,340 sequences per sample. As shown in **Figure 1**, bacterial communities were visualized using principal coordinates analysis (PCoA). PERMANOVA analysis showed that 39.9% of the variance was explained by the presence of caries (R^2^ = 0.399, *p* = 0.001) suggesting that bacterial communities were significantly different between non-carious and carious teeth. However, no differences were observed for alpha diversity metrics (**Figure S2**). Also, surprisingly, no differential features were found when using LEfSe. The oral microbiota of the supragingival dental plaque and carious dentin tissue examined in this study was comprised of 11 phyla as shown in **Figure 2**. Overall, the top 5 phyla were *Firmicutes* (55.99%), *Actinobacteria* (14.28%), *Proteobacteria* (10.05%), *Bacteroidetes* (9.21%), and *Fusobacteria* (8.68%), representing 98.21% of all phyla. A significant increase in the phylum *Actinobacteria* was observed when comparing carious versus non-carious teeth (p < 0.001, **Table S2**). At the genus level, the oral microbiota was composed of 101 bacterial genera: the top 20 genera accounting for 88.89% of the total overall relative abundance (**Table S3**). The top 5 genera were *Veillonella* (21.94%), *Streptococcus* (19.21%), *Lactobacillus* (6.33%)*, Actinomyces* (5.83%), and *Leptotrichia* (5.78%). A significant increase in the relative abundance of *Lactobacillus* (*p* = 0.012), *Actinomyces* (*p* = 0.004), *Prevotella* (*p* = 0.031), *Mitsuokella* (*p* = 0.015), *Campylobacter* (*p* = 0.007), *Selenomonas* (*p* = 0.001), and *Scardovia* (*p* = 0.038) was observed in carious teeth. Conversely, the relative abundance of *Haemophilus* (*p* = 0.008) and *Porphyromonas* (*p* = 0.016) (**Figure 3**, **Table S3**) was reduced in carious teeth. Additionally, *Olsenella* (*p* < 0.001) and *Parascardovia* (*p* = 0.002) were observed only in carious teeth.

### 3.3 Innate immune response of dental pulp tissue exposed to moderate caries

**Figure 4:**
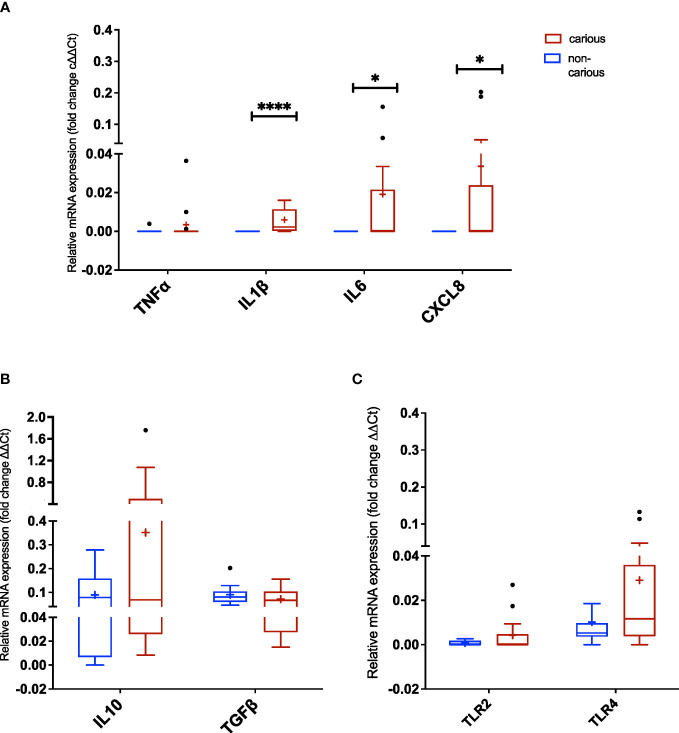
Expression of Toll-like receptors (TLRs) and inflammatory markers in dental pulp tissue non-exposed and exposed to moderate caries. **(A)** TNFα, IL1β, IL6, and CXCL8 (IL8) amplification (three proinflammatory cytokines and one chemokine, respectively). **(B)** IL10 and TGFβ amplification (regulatory cytokines). **(C)** TLR2 and TLR4 amplification. The data are reported as follows: the horizontal solid line within the box represents the median; the position of the cross (+) denotes the average; the box illustrates the results within 25–75 percentiles (interquartile range); the whiskers portray values from 5–95% normalized according to Tukey’s test; the black dots (•) show outliers. Statistical analysis by the Mann-Whitney *U* test, considering significance at p < 0.05 (*p = 0.0407 and ****p < 0.0001).

Next, we assessed the expression of various markers of innate immune response by RT-PCR. mRNA was extracted from the dental pulp tissue of non-carious and carious teeth (n = 14 and n = 17, respectively). As shown in **Figure 4**, two proinflammatory cytokines: IL1β (*p <* 0.0001), IL6 (*p =* 0.041) and one chemokine CXCL8 (*p =* 0.041) were significantly elevated in carious teeth compared to non-carious teeth. No differences were observed for all the other markers (TLR2, TLR4, TFNα, IL10 and TFGβ).

### 3.4 Relationship between oral bacterial microbiota and the innate immune response of dental pulp tissue

**Figure 5:**
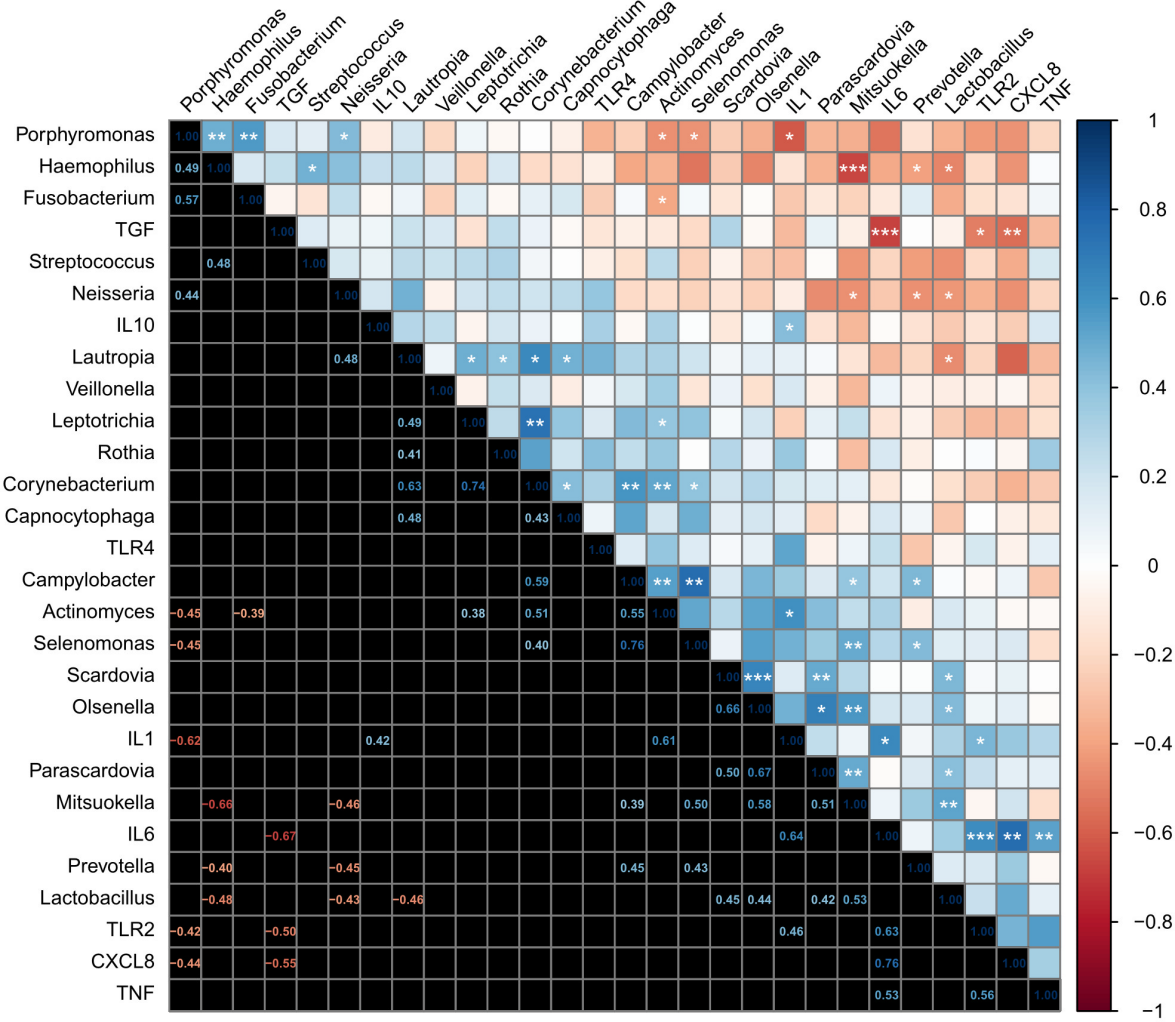
Analysis of the correlation between the oral bacterial microbiota present in moderate caries and the inflammatory markers expressed by the dental pulp tissue. Correlations are shown as a heatmap plot. After subjecting the data to the Shapiro-Wilk test of normality, the results required the application of Spearman’s rank correlation coefficient with the FDR method. Statistical significance was considered at 95% of confidence, with *p ≤ 0.05. Additional p-values are indicated as **p ≤ 0.01; ***p ≤ 0.001.

First, we explored relationships among the top 20 genera. Several positive and negative correlations were observed as shown in **Figure 5**, some of them are listed below. *Lactobacillus* correlated positively *Scardovia* (R^2^ = 0.20, *p =* 0.019), *Olsenella* (R^2^ = 0.19, *p =* 0.023), *Parascardovia* (R^2^ = 0.18, *p =* 0.029), and *Mitsuokella* (R^2^ = 0.28, *p =* 0.005), and negatively with *Haemophilus* (R^2^ = 0.23, *p =* 0.011), *Neisseria* (R^2^ = 0.19, *p =* 0.024) and *Lautropia* (R^2^ = 0.03, *p =* 0.016). Similarly, *Actinomyces* correlated positively with *Leptotrichia* (R^2^ = 0.38, *p =* 0.048), *Corynebacterium* (R^2^ = 0.26, *p =* 0.006), *and Campylobacter* (R^2^ = 0.03, *p =* 0.003); and negatively with *Porphyromonas* (R^2^ = 0.20, *p =* 0.018) and *Fusobacterium* (R^2^ = 0.15, *p =* 0.047). *Prevotella* correlated positively with *Campylobacter* (R^2^ = 0.003, *p =* 0.019) and *Selenomonas* (R^2^ = 0.19, *p =* 0.024); and negatively with *Haemophilus* (R^2^ = 0.16, *p =* 0.038) and *Neisseria* (R^2^ = 0.20, *p =* 0.017). *Mitsuokella* correlated positively with *Campylobacter* (R^2^ = 0.15, *p =* 0.046), *Selenomonas* (R^2^ = 0.25, *p =* 0.007), *Olsenella* (R^2^ = 0.34, *p =* 0.002) and *Parascardovia* (R^2^ = 0.26, *p =* 0.007); and negatively with *Haemophilus* (R^2^ = 0.44, *p <* 0.001), and *Neisseria* (R^2^ = 0.21, *p =* 0.016). *Campylobacter* correlated positively with *Corynebacterium* (R^2^ = 0.35, *p =* 0.001). *Selenomonas* correlated positively with *Corynebacterium* (R^2^ = 0.16, *p =* 0.04) and *Campylobacter* (R^2^ = 0.76, *p =* 0.01). and negatively with *Porphyromonas* (R^2^ = 0.20, *p =* 0.019)*. Olsenella* correlated positively with *Scardovia* (R^2^ = 0.44, *p =* 0.012)*. Haemophilus* correlated positively with *Streptococcus* (R^2^ = 0.23, *p =* 0.012). *Porphyromonas* correlated positively with *Haemophilus* (R^2^ = 0.24, *p =* 0.01), *Fusobacterium* (R^2^ = 0.33, *p =* 0.05) and *Neisseria* (R^2^ = 0.19, *p =* 0.022).

Then, we assessed relationships among markers of innate immune response. TLR2 was positively correlated with IL1β (R^2^ = 0.19, *p =* 0.025), IL6 (R^2^ = 0.19, *p =* 0.001), and negatively with TGFβ (R^2^ = 0.19, *p =* 0.013). Additionally, the expression of IL1β correlated positively with IL10 (R^2^ = 0.18, *p =* 0.040) and IL6 (R^2^ = 0.41, *p <* 0.001). IL6 correlated positively with CXCL8 (R^2^ = 0.58, *p <* 0.001), and TNFα (R^2^ = 0.28, *p =* 0.088); and while correlating negatively with TGFβ (R^2^ = 0.45, *p <* 0.001). CXCL8 correlated negatively with TGFβ (R^2^ = 0.30, *p <* 0.001). Finally, we assessed relationships between the top 20 genera and innate immune response and found a positive correlation between IL1β and *Actinomyces* (R^2^ = 0.37, *p =* 0.046), and negatively *Porphyromonas* (R^2^ = 0.38, *p =* 0.042), (**Figure 5**).

**Figure 6:**
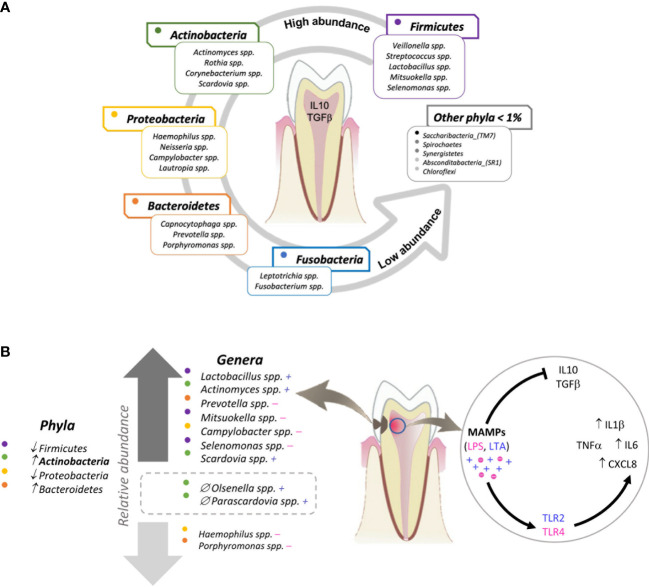
Graphical model of the relationship between the oral bacterial microbiota and the innate immune response of dental pulp tissue in oral health and moderate caries. **(A)** The majority of the microbiome of non-carious teeth (determined with samples taken from the supragingival dental plaque) is represented by 5 phyla and their respective genera. In the presence of these bacterial profiles, dental pulp tissue constitutively expresses the regulatory cytokines IL10 and TGFβ. **(B)** The microbiota of teeth with moderate caries (determined with samples taken from infected dentin tissue) reflects a significant increase in the phylum *Actinobacteria* and the genera *Firmicutes_Lactobacillus*, *Actinobacteria_Actinomyces*, *Bacteroidetes_Prevotella*, *Firmicutes_Mitzuokella*, *Proreobacteria_Campylobacter*, *Firmicutes_Selenomonas*, and *Actinobacteria_Scardovia*, as well as a significant decrease in *Proteobacteria_Haemophilus* and *Proteobacteria_Porphyromonas* compared to the microbiota of non-carious teeth. Additionally, *Actinobacteria*_*Olsenella* and *Actinobacteria_Parascardovia* are present exclusively in moderate caries. Regarding the innate immune response, dental pulp tissue exposed to moderate caries expresses a significant increase in the chemokine CXCL8 and pro-inflammatory cytokines IL6 and IL1β. Each dot indicates a colour assigned to a phylum. The plus (*+*) and minus **(*-*)** signs indicate *Gram*-positive and *Gram-negative bacteria, respectively.* The two genera inside the rectangle drawn with a dotted line are the genera exclusively found in carious teeth (versus non-carious teeth). ↑ increase; ↓ decrease; ∅ exclusive to moderate caries group.

**Figure 6** illustrates the major findings of this study: a wild *ex-vivo* model of dental caries showed that during the natural course of this disease, dental pulp tissue exposed to oral microbiota associated with a moderate stage of dentin caries, overexpressed innate immune proinflammatory cytokines IL1β, IL6 and chemokine CXCL8, thus, contributing to a stage of inflammation driven by the expression of TLRs triggered by different MAMP´s from gram-positive and *Gram*-negative bacteria associated with moderate caries.

## 4 Discussion

This study aimed at understanding changes in the oral microbiota in teeth with a moderate stage of tooth decay. Bacterial taxonomic profiles were correlated with *in situ* expression of markers associated with innate immune response of dental pulp tissue chronically exposed to dentin caries. Caries in the teeth herein analyzed developed under natural conditions. There are some reports on the oral (salivary and dental plaque) microbiota associated with carious teeth (Belda-Ferre et al., 2012; Belstrøm et al., 2014; Belstrøm et al., 2015; Simón-Soro and Mira, 2015; Belstrøm et al., 2017), while others have assessed, *in vitro*, the degree of TLRs and proinflammatory cytokines expression in dental pulp tissue (Couble et al., 2000; Durand et al., 2006; Staquet et al., 2011; Carrouel et al., 2013; Farges and Lyon, 2015; Renard et al., 2016). However, this is the first report, to our knowledge, that evaluates the bacterial microbiome and immune response in teeth a wild *ex-vivo* model of moderate dental caries.

When comparing both groups (non-carious versus carious teeth), we observed differences in the bacterial communities present and the taxonomical composition of the oral microbiome. Individuals with caries showed a significantly higher relative abundance of *Actinobacteria* and *Lactobacillus*, *Actinomyces*, *Prevotella*, *Mitsuokella*, *Selenomonas*, and *Scardovia*, and a lower relative abundance in *Haemophilus* and *Porphyromonas*. Although *Mitsuokella* has not been linked to caries, it is known to play a significant role in the development of periodontal disease. *Mitsuokella dentalis* has been isolated from dental root canals (Haapasalo et al., 1986). Thus, our results showing an increased relative abundance of *Mitsuokella* in the carious group is interesting. On the other hand, a depletion in *Porphyromonas* in the same group agrees with data published by Belstrøm et al. (Belstrøm et al., 2015). Our results are also concordant with a study by Obata and colleagues (Obata et al., 2014) who reported a predominance of *Lactobacillus* and *Prevotella* in the dentin of teeth with caries. In the present study, a tendency to an increase of these two genera was also found in teeth with moderate caries. Another study published by Zheng and colleagues (Zheng et al., 2019) examined the identity of the microbiome (V3-V4 region of the 16S rRNA gene) of deep dentinal caries and its correlation with the inflammation status of caries-induced pulpitis.

Unlike our results, they observed that as the carious lesion progressed, the relative abundance of *Actinobacteria* decreased while *Firmicutes* increased. Interestingly, both studies found a higher relative abundance of the genus *Lactobacillus* in caries. Zheng and colleagues reported a particularly notable increase in this genus in teeth with irreversible symptomatic pulpitis. They demonstrated a strong association between the depth of a carious lesion in the dentin and the presence of reversible or irreversible pulpitis. Likewise, Ferraz Caneppele et al. (Caneppele et al., 2021) described a greater relative abundance of *Lactobacillus*, *Capnocytophaga sputigena*, and *Leptotrichia buccalis* in teeth with deep and symptomatic caries. They concluded that symptomatic caries does not correlate with the bacterial load but instead to the amount of endotoxins, and this is regardless of the area sampled. The present results are also distinct from those reported by Benítez Páez et al. (Benítez-Páez et al., 2014), who identified *Actinomyces* as the most abundant genus in conditions of oral health, and *Porphyromonas*, *Fusobacterium*, *Capnocytophaga*, *Tannerella*, and *Leptotrichia* as the most abundant in a diseased state (Benítez-Páez et al., 2014). According to Simón-Soro et al. (Simón-Soro and Mira, 2015), in the dental plaque on the surface of healthy teeth, the relative abundance of *Fusobacterium* predominates, followed by *Neisseria* and *Streptococcus*.

Since the intra- and intermicrobial interactions are relevant in the pathogenesis of caries, these have been investigated and reported by different authors. For example, Gomez et al. (Gomez et al., 2017) reported a close relationship between *Streptococcus* and *Rothia*, *Actinomyces*, and *Selenomonas* during dental caries; bacteria that have been described as predictive markers of caries. Also associated with dental caries are *Tannerella* and *P. olorum*. An antagonistic relationship between *Streptococcus* and *Fusobacterium* and *Aggregatibacter* was reported by Simón-Soro and colleagues (Simón-Soro et al., 2013) in individuals with caries. These correlations are in partial agreement with our results. Interestingly, the current findings show patterns of correlation between bacterial communities, as previously described by Belstrøm et al. (Belstrøm et al., 2017). They found a significantly higher α-diversity for *Neisseria*, *Haemophilus*, and *Fusobacterium* in saliva samples in the healthy group compared to individuals with a history of having caries. There was an evident clustering between the two groups. Regarding the diversity of the oral microbiota of teeth with caries, our results are concordant with those reported by Belstrøm et al., carried out in Danish subjects. The similarities between these two studies, despite the distinct sites sampled (supragingival dental plaque in our study versus saliva samples in Belstrøm´s study) suggests that determining factors exist regardless of the oral niche sampled. These factors could be related to DNA extraction, PCR amplification, and the hypervariable region used for 16S sequencing. On the other hand, some researchers have argued that it was imperative to compare site-specific data, as we did in the present study (Richards et al., 2017), to gain a better understanding of an irreversible chronic NCD of the oral cavity such as dental caries (Giacaman et al., 2022).

Several authors have assessed the expression of TLR2 and proinflammatory cytokines in distinct dental pulp cells challenged *in vitro* with different components of *Gram*-positive and *Gram*-negative bacteria. In this study, expression profiles of TLR2 and TLR4 and several proinflammatory cytokines were examined as well, but in pulp tissue exposed *in vivo* to modetate caries. Proinflammatory cytokines IL1β and IL6 and the chemokine CXCL8 (IL8) were significantly increased in teeth with caries (versus non-carious teeth). Differential expression of some TLRs and cytokines could be related to the variability in the stage of dental decay. This agrees with findings that differential expression profiles of inflammatory markers are associated with the presence of microbe-associated molecular patterns (MAMPs) from *Gram*-positive and *Gram*-negative bacteria found in caries (Staquet et al., 2011; Farges et al., 2015). Our results are concordant with those reported by various authors. As reported by Farges and colleagues, odontoblast cells stimulated with lipoteichoic acid (LTA) and Pam2CSK4 generated a sharp rise in proinflammatory cytokines, including TNFα, IFNγ, IL1β, IL6, and CXCL8 (Beighton D., 2005). Additionally, they found a similar expression of the regulatory cytokine IL10 between healthy dental pulp tissue and dental pulp tissue exposed to moderate caries; the present study also reports no difference in IL-10. Similarly, Colombini-Ishikiriama et al. (Colombini-Ishikiriama et al., 2020) and Keller et al. (Keller et al., 2010) observed a differential expression of IL1β, IL6, IL8, and TNFα in cultures of pulp fibroblasts of deciduous teeth stimulated with *Escherichia coli*-lipopolysaccharide (EcLPS) or *Enterococcus faecalis*-lipoteichoic acid (EfLTA). However, they also detected a differential expression of IL10.

We acknowledge that this study has several limitations. First, sample size is small. Also, we compared two different types of samples, supragingival dental plaque and carious dentin tissue. The study design is cross-sectional. Although differences were observed between non-carious and carious teeth, our results should be interpreted with caution due to the limitations in statistical power inherent to small sample sizes. We also acknowledge that we did not adjust for confounding variables; when comparing clinical and demographic variables between these two groups, we found no differences in age or sex. This study is, to our knowledge, the first to describe the oral microbiota in Mexican individuals with and without dental caries. The strength of this study is that the oral microbiota associated with caries was evaluated concomitantly with innate immune responses (expression of TLRs and inflammatory markers) of dental pulp tissue exposed *in vivo* to moderate caries.

## 5 Conclusions

A distinct bacterial community structure and a differential expression of innate immune responses were observed in dental caries of Mexican individuals, with 2 genera, *Olsenella* and *Parascardovia*, exclusively found in moderate caries, and IL1β, IL6 and CXCL8 being overexpressed in dental caries. Findings reported here suggest that different pulp cell populations, mostly odontoblasts and fibroblasts, may play a relevant role in the modulation of immune responses of dental pulp tissue chronically exposed to bacteria such as *Lactobacillus*, *Actinomyces* and *Porphyromonas;* driving the stimulation of dental pulp cells by distinct MAMPs, including LTA and lipopolysaccharide (LPS). This modulation may occur *via* TLRs, inducing downstream signaling of proinflammatory cytokines IL1β and IL6 and the chemokine CXCL8. It is imperative to gain insights not only into the microbial profiles associated with dental caries with a moderate degree of progression but also elucidate how the innate immunity of the affected dental pulp tissue responds, to contribute to the development of new strategies to treat caries.

## Data availability statement

The FASTQ reads and datasets presented in this study can be found in online repositories. Repository: SRA accession number: PRJNA842423. The link to the data can be found below: http://www.ncbi.nlm.nih.gov/bioproject/842423.

## Ethics statement

The studies involving human participants were reviewed and approved by Ethics in Research Committee of the Faculty of Medicine of the UNAM, under project approval registry FM/DI/030/SR/2019. The patients/participants provided their written informed consent to participate in this study.

## Author contributions

AG-G contributed with the conceptualization, carried out the investigation, conceived and designed the experiments, and performed the formal analysis and writing-original draft of the manuscript. YL-V contributed with writing-review and editing, writing-original draft, methodology, supervision and validation of results and figures. SP-C contributed to the data curation, technical support for statistical and data analysis and writing-review and editing of the manuscript. MAG contributed to the funding and project administration, conceptualization, and methodology. The original draft was prepared by AG-G and MAG. All authors contributed to the article and approved the submitted version.
