# Supplementary material for: Overexpression of proinflammatory cytokines in dental pulp tissue and distinct bacterial microbiota in carious teeth of Mexican Individuals
Source: Front Cell Infect Microbiol. 2022 Dec 8;12:958722. doi: 10.3389/fcimb.2022.958722 (PMC9772992; doi:10.3389/fcimb.2022.958722)
Supplement: Supplementary file 1 [file Image_1.pdf]

frontiers

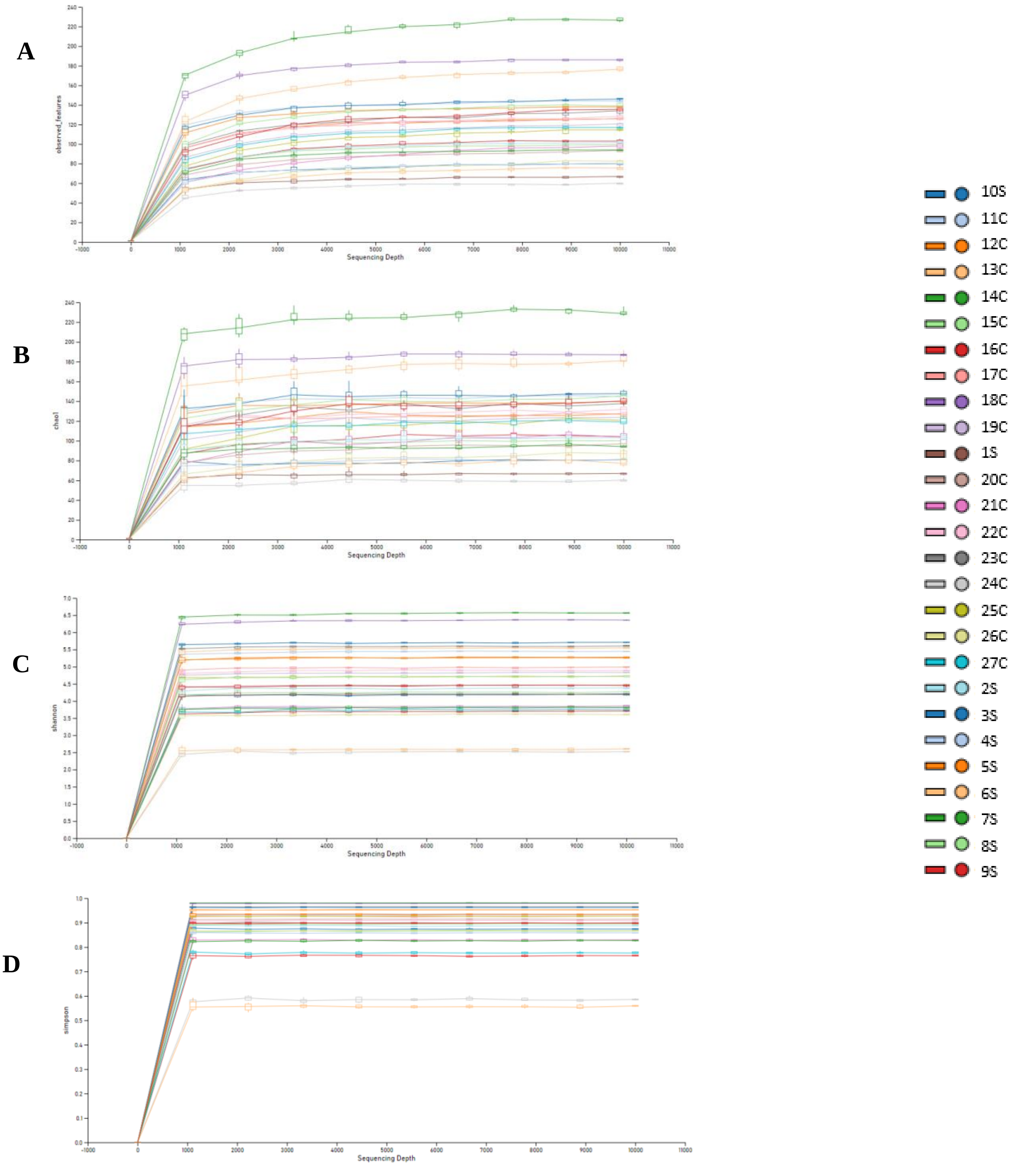

Supplementary figure S1. Rarefaction of amplicon sequence variants (ASVs). Accumulation curves are shown: **(A)** based on the observed species; **(B)** adjusted to the Chao1 richness estimator; **(C)** based on the Shannon diversity index; and **(D)** the Simpson diversity index. The letter *S* refers to samples taken from the supragingival dental plaque of non-carious teeth; the letter *C* refers to samples taken from the infected dentin tissue of carious lesions.
