# Supplementary material for: Overexpression of proinflammatory cytokines in dental pulp tissue and distinct bacterial microbiota in carious teeth of Mexican Individuals
Source: Front Cell Infect Microbiol. 2022 Dec 8;12:958722. doi: 10.3389/fcimb.2022.958722 (PMC9772992; doi:10.3389/fcimb.2022.958722)
Supplement: Supplementary file 2 [file Image_2.pdf]

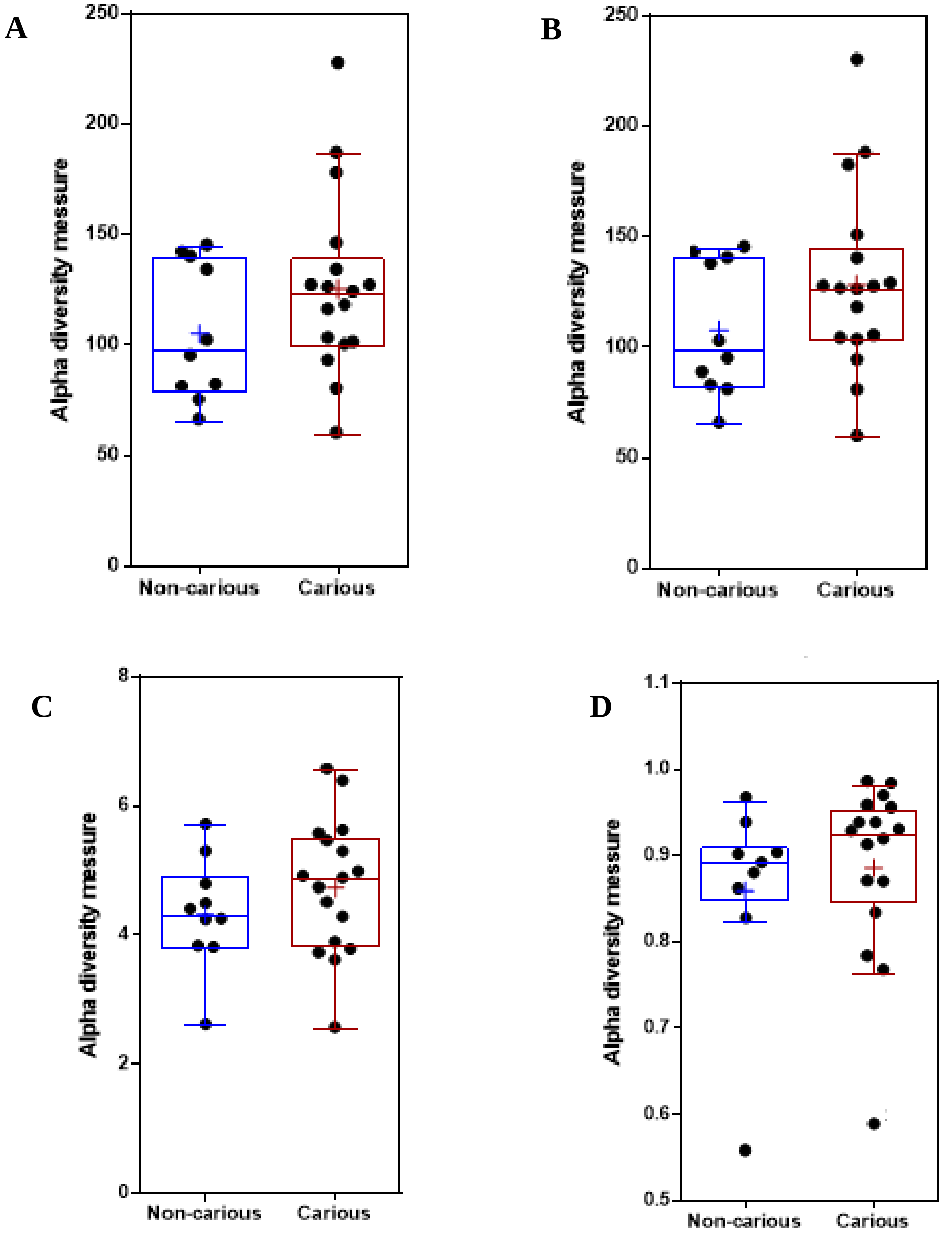

Supplementary figure S2. Alpha diversity analysis. Analysis was conducted based on **(A)** observed ASVs (Me = 97.50 *vs* Me = 123.0, $p = 0.34$); **(B)** chao1 richness estimator (Me = 98.13 *vs* Me = 125.8, $p = 0.30$); **(C)** Shannon diversity index (Me = 4.30 *vs* Me = 4.85, $p = 0.35$); **(D)** Simpson diversity index (Me = 0.89 *vs* Me = 0.92, $p = 0.\ 28$). Data are reported as follows: a horizontal solid line within the box represents the median; the position of the cross (+) denotes the average; the box encompasses the results within 25–75% of the data (interquartile range); the whiskers portray the values from 5–95%, normalized according to Tukey's hinges; the black dots (•) show typical and extreme outlier values. Statistical analysis was performed by the Mann-Whitney *U* test, considering significance at $p \leq 0.05$.
