# Supplementary material for: Overexpression of proinflammatory cytokines in dental pulp tissue and distinct bacterial microbiota in carious teeth of Mexican Individuals
Source: Front Cell Infect Microbiol. 2022 Dec 8;12:958722. doi: 10.3389/fcimb.2022.958722 (PMC9772992; doi:10.3389/fcimb.2022.958722)
Supplement: Supplementary file 3 [file Table_1.docx]

| **Supplementary table S1.** *Oligonucleotide sequences*. | | | | | | |
| --- | --- | --- | --- | --- | --- | --- |
| **Reference** | **Gene** | **Forward 5´- 3´**  **Reverse 5´- 3´** | **Length**  **(nt)** | **Amplicon (bp)** | **Tm °C** | **C-G %** |
| NM_001318787.2 | TLR2 | CAA TGA TGC TGC CAT TCT CAT  ATT ATC TTC CGC AGC TTG CA | 21 20 | 83 | 60  58 | 42.9 45.0 |
| NM_138554.5 | TLR4 | AGT TTC CTG CAA TGG ATC AAG G  CTG CTT ATC TGA AGG TGT TGC AC | 22  23 | 83 | 64  68 | 45.5 47.8 |
| NM_000576.3 | IL1β | GGA TAT GGA GCA ACA AGT GG  ATG TAC CAG TTG GGG AAC TG | 20  20 | 264 | 60  60 | 50.0 50.0 |
| NM_000600.5 | IL6 | GAG CTG TGC AGA TGA GTA CAA  GGA CTG CAG GAA CTC CTT AAA | 21  21 | 190 | 62  62 | 47.6 47.6 |
| NM_000584.4 | CXCL8 (IL8) | TGA AGT GTT GAA GTA GAT TTG C  TGA GAG TGA TTG AGA GTG GA | 22  20 | 224 | 60 58 | 36.0  45.0 |
| NM_000572.3 | IL10 | GCT GGA GA CTT TAA GGG TTA C  GAT GTC TGG GTC TTG GTT CTC | 22  21 | 106 | 66  64 | 50.0  52.4 |
| NM_000594.3 | TNFα | CCT GCC CCA ATC CCT TTA TT  CCC TAA GCC CCC AAT TCT CT | 20  20 | 81 | 60  62 | 50.0 55.0 |
| NM_000660.7 | TGFβ | GCG TGC TAA TGG TGG AAA C  CGG TGA CAT CAA AAG ATA ACC AC | 19  23 | 277 | 58  66 | 52.6  43.5 |
| NM_001101.5 | βactin | TCC CTG GAG AAG AGC TAC G  TAG TTT CGT GGA TGC CAC A | 19  19 | 130 | 60  56 | 57.9  47.4 |
| NM_001256799.2 | GAPDH | GCA CCG TCA AGG CTG AGA AC  TGG TGA AGA CGC CAG TGG A | 20  19 | 138 | 64  60 | 60.0  57.9 |
| Note. TLR2: Toll like receptor 2; TLR4 Toll like receptor 4; IL1β: cytokine 1; IL6: cytokine 6; CXCL8 (IL8): chemokine 8 as well-known as cytokine 8; IL10: cytokine 10; TNFα: tumour necrosis factor alpha; TGFβ: transforming growth factor beta; beta actin; GAPDH: Glyceraldehyde 3-phosphate dehydrogenase; nt: nucleotide; bp: base pair; Tm: melting temperature. | | | | | | |
