# Supplementary material for: Overexpression of proinflammatory cytokines in dental pulp tissue and distinct bacterial microbiota in carious teeth of Mexican Individuals
Source: Front Cell Infect Microbiol. 2022 Dec 8;12:958722. doi: 10.3389/fcimb.2022.958722 (PMC9772992; doi:10.3389/fcimb.2022.958722)
Supplement: Supplementary file 4 [file Table_2.docx]

| **Supplementary table S2.** *Relative abundance of oral bacterial microbiota by phyla.* | | | | |
| --- | --- | --- | --- | --- |
| ***Phyla*** | **Overall** | **Non-carious** | **Carious** | ***p*** |
| *Firmicutes ^a^* | 55.99 | 62.57 | 53.38 | 0.245 |
| *Actinobacteria ^b^* | 14.28 | 4.48 | 18.17 | 0.001^***^ |
| *Proteobacteria ^b^* | 10.05 | 12.63 | 9.03 | 0.547 |
| *Bacteroidetes ^b^* | 9.21 | 9.15 | 9.23 | 0.228 |
| *Fusobacteria ^b^* | 8.68 | 10.62 | 7.91 | 0.725 |
| *Saccharibacteria_(TM7) ^b^* | 0.91 | 0.44 | 1.10 | 0.353 |
| *p__unclassified ^b^* | 0.37 | 0.00 | 0.51 | 0.110 |
| *Spirochaetes ^b^* | 0.31 | 0.05 | 0.41 | 0.210 |
| *Synergistetes ^b^* | 0.12 | 0.01 | 0.17 | 0.271 |
| *Abscondibacteria_(SR1) ^b^* | 0.06 | 0.05 | 0.06 | 0.836 |
| *Chloroflexi ^b^* | 0.02 | 0.00 | 0.03 | 0.104 |
| *Note.* Data are expressed as a percentage.  ^a^ The parametric Student’s *t*-test was applied for normal distribution of data, assuming unequal variances.  ^b^ When the data did not meet the assumptions of normality, the non-parametric Mann-Whitney *U* test for independent samples was applied.  Statistical significance was considered at 95% of confidence, (*p* ≤ 0.05). *** *p* < 0.001. | | | | |
