# Supplementary material for: Overexpression of proinflammatory cytokines in dental pulp tissue and distinct bacterial microbiota in carious teeth of Mexican Individuals
Source: Front Cell Infect Microbiol. 2022 Dec 8;12:958722. doi: 10.3389/fcimb.2022.958722 (PMC9772992; doi:10.3389/fcimb.2022.958722)
Supplement: Supplementary file 5 [file Table_3.docx]

| **Supplementary table S3.** *Relative abundance of oral bacterial microbiota by genus.* | | | | |
| --- | --- | --- | --- | --- |
| ***Genus*** | **Overall** | **Non-carious** | **Carious** | ***p*** |
| *p__Firmicutes;g__Veillonella* | 21.94 | 28.08 | 19.10 | 0.482 |
| *p__Firmicutes;g__Streptococcus* | 19.21 | 30.40 | 14.42 | 0.120 |
| *p__Firmicutes;g__Lactobacillus* | 6.33 | 0.23 | 8.63 | 0.012^*^ |
| *p__Actinobacteria;g__Actinomyces* | 5.83 | 2.28 | 7.13 | 0.004^**^ |
| *p__Fusobacteria;g__Leptotrichia* | 5.78 | 6.46 | 5.41 | 0.880 |
| *p__Bacteroidetes;g__Capnocytophaga* | 3.27 | 2.81 | 3.39 | 0.451 |
| *p__Bacteroidetes;g__Prevotella* | 3.27 | 1.61 | 3.86 | 0.031^*^ |
| *p__Fusobacteria;g__Fusobacterium* | 3.02 | 4.16 | 2.51 | 0.422 |
| *p__Firmicutes;g__Mitsuokella* | 2.65 | 0.05 | 3.63 | 0.015^*^ |
| *p__Proteobacteria;g__Haemophilus* | 2.36 | 5.60 | 1.03 | 0.008^**^ |
| *p__Actinobacteria;g__Rothia* | 2.09 | 0.69 | 2.61 | 0.120 |
| *p__Proteobacteria;g__Neisseria* | 1.82 | 4.01 | 0.92 | 0.071 |
| *p__Proteobacteria;g__Campylobacter* | 1.73 | 0.81 | 2.07 | 0.007^**^ |
| *p__Firmicutes;g__Selenomonas* | 1.72 | 0.46 | 2.19 | 0.001^**^ |
| *p__Proteobacteria;g__Lautropia* | 1.50 | 0.53 | 1.86 | 0.498 |
| *p__Actinobacteria;g__Olsenella* | 1.49 | 0.00 | 2.05 | < 0.001^***^ |
| *p__Bacteroidetes;g__Porphyromonas* | 1.46 | 3.67 | 0.56 | 0.016^*^ |
| *p__Actinobacteria;g__Parascardovia* | 1.18 | 0.00 | 1.62 | 0.002^**^ |
| *p__Actinobacteria;g__Corynebacterium* | 1.15 | 0.48 | 1.39 | 0.144 |
| *p__Actinobacteria;g__Scardovia* | 1.09 | 0.55 | 1.28 | 0.038^*^ |
| *Note.*  Data are expressed as a percentage. Since the data did not meet the assumptions of normality, the non-parametric Mann-Whitney *U* test for independent samples was applied. Statistical significance was considered at 95% of confidence, with **p* ≤ 0.05. Other *p*-values are indicated: ***p* < 0.01 and ****p* < 0.001. | | | | |
